# Functional morphometry: non-invasive estimation of the alveolar surface area in extremely preterm infants

**DOI:** 10.1038/s41390-023-02597-z

**Published:** 2023-04-12

**Authors:** Emma E. Williams, J. Gareth Jones, Donald McCurnin, Mario Rüdiger, Mahesh Nanjundappa, Anne Greenough, Theodore Dassios

**Affiliations:** 1 Women and Children’s Health, School of Life Course Sciences, Faculty of Life Sciences and Medicine, King’s College London, London, UK; 2 Cambridge University Clinical School, Hills Rd, Cambridge, UK; 3 Division of Neonatology, Department of Pediatrics, University of Texas Health, San Antonio, TX USA; 4 Neonatology and Pediatric Critical Care Medicine, Department of Pediatrics, Medizinische Fakultät, Carl Gustav Carus, TU Dresden, Dresden, Germany; 5 Saxony Center for Feto/Neonatal Health, Medizinische Fakultät, TU Dresden, Dresden, Germany; 6 Neonatal Intensive Care Centre, King’s College Hospital NHS Foundation Trust, London, UK; 7 National Institute for Health Research (NIHR) Biomedical Research Centre based at Guy’s and St Thomas’ NHS Foundation Trust and King’s College London, London, UK

## Abstract

**Background:**

The main pathophysiologic characteristic of chronic respiratory disease following extremely premature birth is arrested alveolar growth, which translates to a smaller alveolar surface area (S_A_). We aimed to use non-invasive measurements to estimate the S_A_ in extremely preterm infants.

**Methods:**

Paired measurements of the fraction of inspired oxygen and transcutaneous oxygen saturation were used to calculate the ventilation/perfusion ratio, which was translated to S_A_ using Fick’s law of diffusion. The S_A_ was then adjusted using volumetric capnography.

**Results:**

Thirty infants with a median (range) gestational age of 26.3 (22.9–27.9) weeks were studied. The median (range) adjusted S_A_ was 647.9 (316.4–902.7) cm^2^. The adjusted S_A_ was lower in the infants who required home oxygen [637.7 (323.5–837.5) cm^2^] compared to those who did not [799.1 (444.2–902.7) cm^2^, *p* = 0.016]. In predicting the need for supplemental home oxygen, the adjusted S_A_ had an area under the receiver operator characteristic curve of 0.815 (*p* = 0.017). An adjusted S_A_ ≥688.6 cm^2^ had 86% sensitivity and 77% specificity in predicting the need for supplemental home oxygen.

**Conclusions:**

The alveolar surface area can be estimated non-invasively in extremely preterm infants. The adjusted alveolar surface area has the potential to predict the subsequent need for discharge home on supplemental oxygen.

**Impact:**

- We describe a novel biomarker of respiratory disease following extremely preterm birth.
- The adjusted alveolar surface area index was derived by non-invasive measurements of the ventilation/perfusion ratio and adjusted by concurrent measurements of volumetric capnography.
- The adjusted alveolar surface area was markedly reduced in extremely preterm infants studied at 7 days of life and could predict the need for discharge home on supplemental oxygen.
- This method could be used at the bedside to estimate the alveolar surface area and provide an index of the severity of lung disease, and assist in monitoring, clinical management and prognosis.

## Introduction

Extremely preterm birth, occurring before 28 completed weeks of gestation, is almost universally associated with respiratory disease,^1^ which in the chronic phase can evolve into a multifactorial disorder called bronchopulmonary dysplasia (BPD).^2,3^ Important recent advances in neonatal care have decreased the threshold of survival to 22 weeks of gestation, but the incidence of BPD has increased, as more preterm infants survive and the ones who survive are more immature.^4^ The diagnosis of BPD has lifelong consequences. Respiratory services are now providing care to the new patient population of ‘BPD adults’ who have severe persistent respiratory complications, associated abnormal neurodevelopment and impaired quality of life.^5^ Despite the clinical significance, BPD has been historically diagnosed as a binary condition,^6^ was then categorised to mild, moderate and severe^7^ and more recently classified into four categories of severity.^8^ This binary or categorical classification, however, fails to capture the granularity of the disease spectrum.^9^ There is, thus, a pressing clinical need for reliable biomarkers of BPD that could assist in quantifying severity, personalised monitoring, early decision making and family counselling.

The cardinal pathological process in BPD is the arrest of alveolar development which leads to a simplification of the lung structure with fewer and larger alveolar sacs.^10^ This translates to a smaller alveolar surface area (S_A_) which, in later life, can become a limiting factor, manifesting as decreased respiratory reserves, limited exercise capacity and functional limitations affecting everyday life.^11^ Although the S_A_ would be an ideal biomarker to quantify and monitor BPD, it cannot be measured in newborn infants as the gold standard measurement method of stereological morphometry can only be performed post-mortem, or using functional tests such as the diffusing capacity for carbon monoxide which require volitional manoeuvres, reliably performed only by older children.

We have recently described an alternative method to measure the S_A_ in living individuals by functional morphometry, which is the non-invasive estimation of the S_A_ based on paired measurements of the fraction of inspired oxygen and transcutaneous oxygen saturation based on Fick’s first law of diffusion^12^ and we validated the method using gold standard stereology in a model of extreme prematurity using non-human primates, preterm baboons.^13^

In this study, we aimed to use functional morphometry to measure the S_A_ in extremely preterm human infants. We also aimed to explore whether the S_A_ was related to respiratory outcomes of extreme prematurity such as the need for supplemental home oxygen and hence had the potential to predict chronic respiratory morbidity.

## Methods

### Subjects

Extremely preterm infants (born before 28 completed weeks of gestation) were recruited between 1 October 2020 and 31 January 2022 at the Neonatal Intensive Care Unit, King’s College Hospital NHS Foundation Trust, London, UK. Recruited infants were studied at 1 week after birth and the study continued for 6 months after the recruitment of the last infant to allow for the collection of the outcome data. The time point was selected as it has been demonstrated that positive pressure ventilation at 7 days after birth was 99% sensitive in predicting the later development of BPD.^14^ Infants were ventilated by volume-targeted ventilation using the SLE 6000 neonatal ventilator (SLE, Croydon, UK) and were intubated with Cole’s shouldered endotracheal tubes, which minimise the expiratory leak.^15^ The study was approved by the Brighton and Sussex Research Ethics Committee, UK [REC 20/PR/0299]. Parents of eligible infants were approached and the infants were recruited after written informed consent. The study was registered on clinicaltrials.gov [NCT 04936477].

### Functional morphometry

As the gold standard method of stereological morphometry to measure the S_A_ can only be applied post-mortem,^16^ estimation of the S_A_ was undertaken by functional morphometry. This method utilises non-invasive measurements of the ventilation-perfusion ratio (V_A_/Q) and Fick’s First law of diffusion, after adjusting for pulmonary perfusion, thickness of the respiratory membrane and the alveolar-arterial gradient (AaG).^12^ We have previously used traditional stereological morphometry to validate this method, using a non-human primate facility of preterm baboons.^13^ The predictive equation that was utilised to estimate the S_A_ was:^13^

$${{{{{\mathrm{S}}}}}}_{{{{{\mathrm{A}}}}}} = 2139 \,{{\times}} \left( {{{{{{\mathrm{V}}}}}}_{{{{{\mathrm{A}}}}}}/{{{{{\mathrm{Q}}}}}}} \right) - 75\left( {{{{{{{{\mathrm{cm}}}}}}}}^2} \right)$$

Where, S_A_ = surface area in cm^2^, and V_A_/Q = ventilation/perfusion ratio.

### Ventilation/perfusion ratio

Inequalities of ventilation and perfusion in non-homogenous lung disease can contribute low, normal or high V_A_/Q ratios and the resulting effect on oxygenation can be measured non-invasively using the oxyhaemoglobin dissociation curve.^17,18^ All infants were nursed supine for consistency, as regional pulmonary blood flow and the delivery of ventilation to different zones of the lungs can vary depending on position.^19^ To calculate the V_A_/Q,^20^ three to five paired measurements of transcutaneous oxygen saturation (SpO_2_) and fraction of inspired oxygen (FiO_2_) were recorded by altering the provided FiO_2_ so that the SpO_2_ varied within a predefined range of 86–100% for 5–10 min. A Nellcor neonatal pulse oximetry probe (Medtronic, Minneapolis) was used. A previously published computer software was used.^21,22^ The sigmoid oxyhaemoglobin dissociation curve depicts the partial pressure of oxygen (P_I_O_2_) in kPa on the *x*-axis and the oxygen saturation of haemoglobin (%) on the *y*-axis; with the shifting of the curve to the right equating to a lower V_A_/Q.^13,20^ The computer software superimposed a best-fit oxyhaemoglobin dissociation reference curve to individual infant data, thus giving a calculated value of V_A_/Q.^23^ The software used the fetal curve as a reference and incorporated the concurrent haemoglobin value (mg/dL) measured by blood gas analysis.

### Cardiac measurements

Targeted echocardiography (ECHO) was concurrently performed by a certified neonatal clinician utilising the Philips Affiniti 50G ultrasound system (Philips, Amsterdam, NL). The following parameters were measured: presence and direction of any intracardiac shunting, any patent ductus arteriosus (PDA) (and if present, the size and direction of flow), pulmonary to systemic flow ratio (Qp/Qs) and left atrial (LA) to aortic ratio. All ECHO findings were reviewed by an external paediatric cardiology consultant for quality control. The ECHO measurements were performed to demonstrate that the pulmonary blood flow (Qp) and cardiac output (CO) were comparable to the parameters of the baboon model and confirm that the regression equation was transferable to human data.^13^ The ECHO was also performed to confirm that the direction of the ductal flow was left-to-right, as a predominantly right-to-left ductal flow would cause hypoxaemia and affect the calculation of the V_A_/Q. The AaG was also calculated to demonstrate that the regression equation was transferable to human data.^24^

### Respiratory dead space

An NM3 mainstream capnograph with a combined flow sensor (Philips Respironics, Connecticut) and a dead space of less than 1 mL^25^ was incorporated into the ventilator circuit between the endotracheal tube and the ventilator circuit for 10 min to collect expired carbon dioxide (CO_2_) and volume data used for the construction of volumetric capnograms and the calculation of the physiological and alveolar dead space.^26^ The measurement of exhaled CO_2_ was only performed in the infants who were invasively ventilated at the time of the study. For consistency, only ventilator inflations as opposed to spontaneous infant breaths were analysed. The start of expiration was defined as the start of negative flow with the end of expiration corresponding to the end of negative flow. No time delay was observed between the CO_2_ and flow signals, with the maximal end-tidal CO_2_ aligning with the end of expiration as determined by the flow wave.

Flow was integrated over time to calculate the expiratory tidal volume for each expiration. The expiratory tidal volume with the corresponding CO_2_ was combined to calculate the mean CO_2_ of mixed expired air:

$${{{{{\mathrm{P}}}}}}_{{{{{{\mathrm{emean}}}}}}}{{{{{{\mathrm{CO}}}}}}_2} = \frac{{{\int}_{{{{{{\mathrm{V}}}}}} = {{{{{\mathrm{Vend}}}}}}_{{{{{{\mathrm{insp}}}}}}}}^{{{{{{\mathrm{Vend}}}}}}_{\exp }} {{{{{{\mathrm{PCO}}}}}}_2 \cdot {{{{{\mathrm{dV}}}}}}} }}{{{{{{{\mathrm{V}}}}}}_{{{{{{\mathrm{te}}}}}}}}}$$

Where, P_emean_CO_2_ is the mean CO_2_ of the mixed expired air in mmHg, V_te_ is the expired tidal volume in mL, PCO_2_ is the partial pressure of CO_2_ in the blood in mmHg, Vend_exp_ is the volume at the end of expiration and Vend_insp_ is the volume at the end of inspiration.^27^

The dead space was calculated from the Enghoff modification of the Bohr equation.^28^ The modified Bohr–Enghoff equation was chosen as it has been shown that it can provide dead space measurements regardless of the shape of the volumetric capnogram and the presence of an alveolar plateau.^29,30^

The physiological dead space (V_Dphys_) (mL) was calculated as follows:

$${{{{{{{\mathrm{V}}}}}}}}_{{{{{{{{\mathrm{Dphys}}}}}}}}} = {{{{{{{\mathrm{V}}}}}}}}_{{{{{{{{\mathrm{te}}}}}}}}}\,{{\times}}\left( {{{{{{{{\mathrm{P}}}}}}}}_{{{{{{{\mathrm{a}}}}}}}}{{{{{{{\mathrm{CO}}}}}}}}_2 - {{{{{{{\mathrm{P}}}}}}}}_{{{{{{{{\mathrm{emean}}}}}}}}}{{{{{{{\mathrm{CO}}}}}}}}_2} \right)/{{{{{{{\mathrm{P}}}}}}}}_{{{{{{{\mathrm{a}}}}}}}}{{{{{{{\mathrm{CO}}}}}}}}_2$$

The anatomical dead space (V_Dana_) (mL) was calculated as follows:

$${{{{{{{\mathrm{V}}}}}}}}_{{{{{{{{\mathrm{Dana}}}}}}}}} = {{{{{{{\mathrm{V}}}}}}}}_{{{{{{{{\mathrm{te}}}}}}}}}\,{{\times}}\left( {{{{{{{{\mathrm{P}}}}}}}}_{{{{{{{{\mathrm{Et}}}}}}}}}{{{{{{{\mathrm{CO}}}}}}}}_2 - {{{{{{{\mathrm{P}}}}}}}}_{{{{{{{{\mathrm{emean}}}}}}}}}{{{{{{{\mathrm{CO}}}}}}}}_2} \right)/{{{{{{{\mathrm{P}}}}}}}}_{{{{{{{{\mathrm{Et}}}}}}}}}{{{{{{{\mathrm{CO}}}}}}}}_2$$

The alveolar dead space (V_Dalv_) (mL) was calculated as follows:

$${{{{{{{\mathrm{V}}}}}}}}_{{{{{{{{\mathrm{Dalv}}}}}}}}} = {{{{{{{\mathrm{V}}}}}}}}_{{{{{{{{\mathrm{Dphys}}}}}}}}} - {{{{{{{\mathrm{V}}}}}}}}_{{{{{{{{\mathrm{Dana}}}}}}}}}$$

Finally, the alveolar tidal volume (Vt_ALV_) (mL) was calculated as follows:

$${{{{{{{\mathrm{Vt}}}}}}}}_{{{{{{{{\mathrm{ALV}}}}}}}}} = {{{{{{{\mathrm{V}}}}}}}}_{{{{{{{{\mathrm{te}}}}}}}}} - {{{{{{{\mathrm{V}}}}}}}}_{{{{{{{{\mathrm{Dana}}}}}}}}}$$

Where, V_te_ is the expired tidal volume in mL, P_a_CO_2_ is the partial arterial pressure of CO_2_ in mmHg, P_Et_CO_2_ is the maximal end-tidal CO_2_ in mmHg.^27^

### Adjusted S_A_ index

**Fig. 1:**
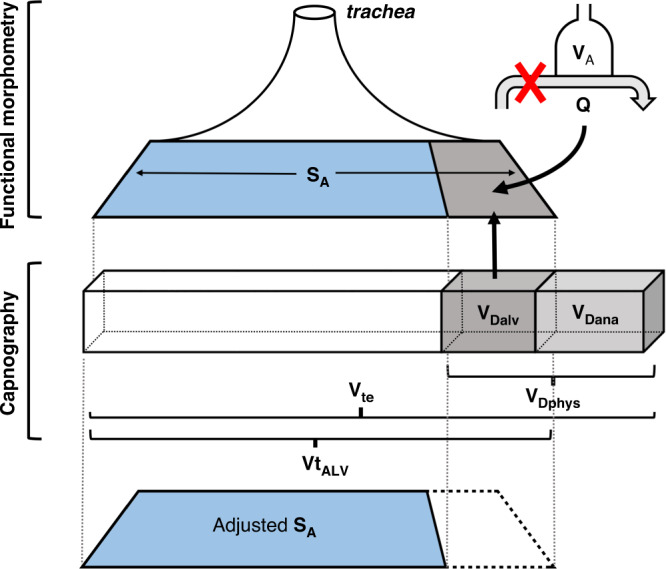
Schematic depiction of the calculation of the adjusted alveolar surface area from the alveolar surface area (S_A_). The S_A_ was derived from the V_A_/Q measurements using functional morphometry. The alveolar dead space (V_Dalv_) was calculated by subtraction of the anatomical dead space (V_Dana_) from the physiological dead space (V_Dphys_). The alveolar tidal volume (Vt_ALV_) was calculated by deducting the V_Dana_ from the expired tidal volume (V_te_). The part of the total S_A_ that corresponded to the V_Dalv_ was extracted from the total S_A_ to derive the adjusted S_A_.

The histological measurements used to calculate the S_A_ correspond to all aerated/ventilated lung units, including those which are not perfused (alveolar dead space).^13^ To give a more precise index of the functioning S_A_ which is taking part in gas exchange, the alveolar dead space was deducted from the S_A_ to derive an adjusted S_A_ index (Fig. 1). The adjusted S_A_ index was calculated as follows:

$${{{{{{{\mathrm{Adjusted}}}}}}}}\,{{{{{{{\mathrm{S}}}}}}}}_{{{{{{{\mathrm{A}}}}}}}}\,{{{{{{{\mathrm{index}}}}}}}}\left( {{{{{{{{\mathrm{cm}}}}}}}}^2} \right) = {{{{{{{\mathrm{S}}}}}}}}_{{{{{{{\mathrm{A}}}}}}}} - \left( {{{{{{{{\mathrm{S}}}}}}}}_{{{{{{{\mathrm{A}}}}}}}} \ast \left( {{{{{{{{\mathrm{V}}}}}}}}_{{{{{{{{\mathrm{Dalv}}}}}}}}}/{{{{{{{\mathrm{Vt}}}}}}}}_{{{{{{{{\mathrm{ALV}}}}}}}}}} \right)} \right]$$

### Outcomes

The primary outcome was the requirement for supplemental home oxygen on discharge from neonatal care.^31,32^ This outcome was selected as it has been previously demonstrated that infants with BPD discharged on supplemental oxygen exhibit higher rates of rehospitalisation, require greater use of respiratory medications and have more frequent attendances to respiratory specialists at 24 months of age compared to infants with BPD who were not discharged on supplemental oxygen.^31^ The clinicians caring for the included infants were not aware of the study results at any point up to discharge from neonatal care. Secondary outcomes were the severity of BPD at 36 weeks postmenstrual age,^7^ the duration of mechanical ventilation, the duration of supplemental oxygen during inpatient stay and neonatal mortality prior to discharge.

### Sample size calculation

Since only a small case series of stereological morphometry has been published reporting the S_A_ in extremely preterm infants, and as V_A_/Q is the major determinant of S_A_ using functional morphometry, the sample size calculation was based on previously reported measurements of V_A_/Q. A clinically significant difference in V_A_/Q of 0.21 was observed between premature infants with severe BPD requiring supplemental oxygen at 36 weeks PMA (V_A_/Q = 0.38) compared to infants with severe BPD who did not (V_A_/Q = 0.59),^9^ and using a standard deviation in V_A_/Q of 0.21,^33^ a sample of 30 infants was required to detect such a difference in V_A_/Q with 80% power at a significance level of 5%.

### Statistical analysis

The data were tested for normality using the Shapiro–Wilk test and found to be non-normally distributed. Data were presented as median and range. The Mann–Whitney U test was performed to determine if differences in S_A_ in infants that were discharged on supplemental oxygen versus the ones that did not and in infants with severe BPD versus the ones with mild/moderate BPD were statistically significant. Spearman’s Rho correlation analysis was utilised to assess the strength of relationships between the S_A_ and the gestational age, birth weight, birth weight *z*-score, duration of mechanical ventilation and duration of inpatient oxygen therapy. The relationship of the S_A_ with the duration of inpatient supplemental oxygen was graphically depicted with linear regression analysis. The ability of the adjusted S_A_ index to predict the need for supplemental home oxygen at discharge was assessed with receiver operating characteristic (ROC) curve analysis. Statistical analysis was performed using SPSS software version 27 (SPSS Inc., Chicago, IL).

## Results

### Characteristics of the cohort

Thirty infants were recruited with a median (range) gestational age of 26.3 (22.9–27.9) weeks, a birth weight of 805 (515–1165) g and were studied at a postnatal age of 7 (5–9) days. Twenty infants had a complete course of antenatal corticosteroids and all infants received at least one dose of surfactant. Twenty-six infants received invasive ventilation during the study. The targeted tidal volume was 6.0 (5.0–6.8) mL/kg [Table 1]. Seventeen infants were medically treated for a PDA and two infants had ligation of the duct. Five infants were diagnosed with a grade III/IV intraventricular haemorrhage and no infant developed periventricular leucomalacia. Three infants required surgical intervention for necrotising enterocolitis and five infants required treatment for retinopathy. Ten infants received postnatal corticosteroids.

**Table 1:** Characteristics of the study population.

Gestational age (weeks)	26.3 (22.9–27.9)
Birth weight (g)	805 (515–1165)
Birth weight *z*-score	−0.30 (−2.2 to 0.99)
Male gender	18 (60.0)
Day of life at study	7 (5–9)
Postmenstrual age at study (weeks)	27.1 (23.4–28.9)
Weight at study (g)	822 (450–1155)
Duration of mechanical ventilation (days)	39 (3–153)
Duration of non-invasive ventilation (days)	55 (23–134)
Total length of stay (days)	131 (61–180)
Postmenstrual age at discharge (weeks)	44.1 (35.7–53.4)
Weight at discharge (g)	3845 (2170–5980)
Survival to discharge	28 (93.3)
Severe bronchopulmonary dysplasia or death	20 (66.7)
Home oxygen requirement	18 (60.0)

Median (range) or number (%).

### Cardiac parameters

Twenty-eight infants had a PDA at the time of the study. All but one infant exhibited a left-to-right flow across the ductus arteriosus, and one had a bidirectional flow. The median (range) pulmonary blood flow (Qp) was 350 (200–1050) mL/min [Table 2].

**Table 2:** Echocardiographic parameters.

Left atrial to aortic ratio	1.6 (1.0–2.0)
Ductus size (mm)	1.7 (1.0–3.1)
Cardiac output (mL/min)	200 (100–500)
Cardiac output (mL/min/kg)	268 (130–510)
Pulmonary perfusion (mL/min)	350 (200–1050)
Pulmonary perfusion (mL/min/kg)	394 (192–1029)

Median (range).

### Respiratory dead space

The median (range) P_a_CO_2_ was 45.0 (29.9–81.8) mmHg and the corresponding P_Et_CO_2_ was 29.0 (13.9–41.6) mmHg. The physiological dead space was 5.8 (3.9–9.7) mL/kg, anatomical dead space 5.1 (3.6–7.4) mL/kg and alveolar dead space 0.64 (0.32–2.33) mL/kg. The dead space to tidal volume ratio was 0.82 (0.71–0.95). The alveolar tidal volume was 1.90 (0.54–6.33) mL/kg and the alveolar dead space: alveolar tidal volume ratio was 0.37 (0.21–0.63).

### Functional morphometry and adjusted S_A_ index

The median (range) Hb at the time of the study was 12.5 (10.7–15.2) g/dL. The V_A_/Q was 0.52 (0.39–0.61). The alveolar surface area was 1026.6 (759.2–1229.8) cm^2^ and the adjusted S_A_ was 647.9 (316.4–902.7) cm^2^. There was no significant difference in the S_A_ (*p* = 0.721) or adjusted S_A_ index (*p* = 0.551) between male and female infants. The S_A_ did not differ significantly between invasively ventilated infants [1037.3 (759.2–1208.4) cm^2^] and infants on non-invasive support [1069.4 (1015.9–1229.8) cm^2^, *p* = 0.285]. The adjusted S_A_ in the one infant with bidirectional flow through the PDA was 785.0 cm^2^ and in the infant with a right-to-left shunt was 444.2 cm^2^.

### Outcomes

Two infants died prior to discharge from the neonatal intensive care unit. All infants surviving to 36 weeks postmenstrual age were diagnosed with BPD: two infants had mild, eight infants had moderate and the remaining 18 infants had severe BPD. The median duration of inpatient supplemental oxygen was 101 (40–171) days. Eighteen infants were discharged home from neonatal care requiring supplemental oxygen with an oxygen flow of 100 (10–500) mL/min.

**Fig. 2:**
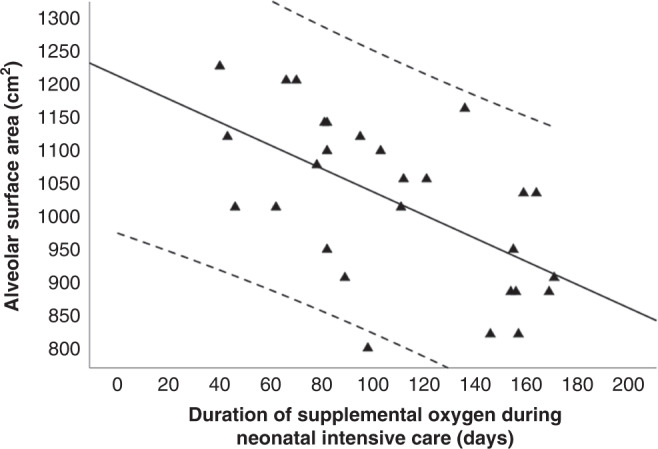
Linear regression of the alveolar surface area (S_A_) and the duration of supplemental oxygen during inpatient stay. The regression line (*R*^2^ = 0.348, *p* < 0.001) is shown with 95% confidence intervals [dashed lines].

The S_A_ was negatively correlated with the duration of mechanical ventilation (*r* = −0.417, *p* = 0.027) and the duration of inpatient supplemental oxygen (*r* = −0.580, *p* = 0.001, Fig. 2). There were no significant correlations of the S_A_ with the gestational age (*r* = 0.125, *p* = 0.509), birth weight (*r* = 0.128, *p* = 0.499) or birth weight *z*-score (*r* = 0.114, *p* = 0.556). The S_A_ did not differ significantly between infants who required supplemental home oxygen [983.8 (823.4–1208.4) cm^2^] and the ones who did not [1091.8 (802.0–1229.8) cm^2^, *p* = 0.111].

The adjusted S_A_ index had a negative correlation with the duration of supplemental oxygen as inpatient (*r* = −0.504, *p* = 0.012). There were no significant correlations of the adjusted S_A_ index with gestational age (*r* = 0.331, *p* = 0.098), birth weight (*r* = 0.335, *p* = 0.094) or birth weight *z*-score (*r* = 0.071, *p* = 0.737). The adjusted S_A_ index was not significantly different in infants with severe compared to mild or moderate BPD (*p* = 0.417), nor was it associated with the duration of invasive mechanical ventilation (*r* = −0.301, *p* = 0.154).

Home oxygen requirement at discharge was not significantly associated with gestational age (*p* = 0.515), birth weight (*p* = 0.428), birth weight *z*-score (*p* = 0.083), antenatal corticosteroid exposure (*p* = 0.839), postnatal corticosteroid therapy (*p* = 0.061), V_A_/Q (*p* = 0.111) or alveolar dead space (*p* = 0.804).

**Fig. 3:**
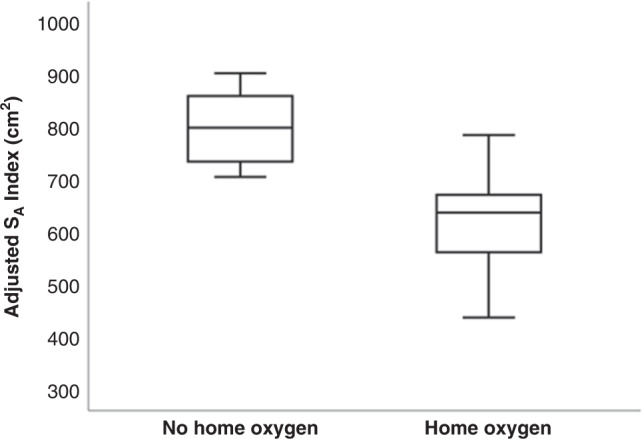
The horizontal line of the boxes depict the 25th, 50th and 75th percentiles and the whiskers the maximum and minimum values. Box plot of the adjusted S_A_ index in those who required home oxygen at discharge and those who did not.

The adjusted S_A_ index was lower in infants who required supplemental home oxygen [637.7(323.5–837.5) cm^2^] compared to the ones who did not [799.1(444.2–902.7) cm^2^, *p* = 0.016) (Fig. 3). In predicting the need for supplemental home oxygen the adjusted S_A_ index had an area under the ROC curve of 0.815 (*p* = 0.017). An adjusted S_A_ index greater or equal to 688.6 cm^2^ had 86% sensitivity and 77% specificity in predicting the need for supplemental home oxygen. The positive predictive value was 92% and the negative predictive value was 55%.

## Discussion

We have demonstrated that the non-invasive method of functional morphometry can be used to estimate the alveolar surface area in extremely preterm infants. The derived biomarker of the adjusted S_A_ predicted the requirement for supplemental home oxygen on discharge from neonatal care with moderate sensitivity and specificity.

Non-invasive measurements of V_A_/Q have been reported to have a moderate ability to predict disease severity in infants with BPD at 36 weeks postmenstrual age with an area under the curve of 0.83.^34^ In addition, V_A_/Q has been utilised to differentiate infants with severe BPD who require supplemental oxygen at 36 weeks PMA against those who do not—suggesting that V_A_/Q is a sensitive marker for describing the impairment in oxygenation associated with lung disease of prematurity.^9^ A longer duration of oxygen therapy during neonatal care in the current study was associated with a reduced alveolar surface area. This finding is in agreement with a study that used high-resolution computed tomography and reported that prolonged oxygen therapy during neonatal care was associated with long-term abnormalities in the lung parenchyma.^35^ In the current study, the V_A_/Q was further corrected by deducting the alveolar dead space to more accurately include areas of the gas-exchanging membrane which are both aerated and perfused and the derived new index was measured as early as at 1 week of postnatal life.

Previous studies have used post-mortem stereological morphometry to calculate the alveolar surface area in infants born prematurely but have included very few infants and with a wide range of postnatal ages. A smaller alveolar surface area at 0.3–1.0 m^2^ has been demonstrated in eight infants with BPD studied between 2 and 28 months of age compared to 1.7–5.8 m^2^ in term-born controls.^36^ Our study reports values which are lower than previously described; however, the infants included in our cohort were all born extremely preterm, including some born at 22 weeks of gestation, and their alveolar surface area was calculated at an earlier time point (7 days). Since it is known that the lung volume and total alveolar number increase with advancing maturity, the lower values of the alveolar surface area in our study are not an unexpected finding.^37^ It should also be noted that the alveolar surface area was calculated based on a regression model which used the results only from the left lung in the premature baboon model;^13^ the adjusted S_A_ thus cannot be seen as a true measurement of the total alveolar surface area but rather as a sensitive approximating index.

Based on the pathophysiology of BPD and the corresponding decrease in the alveolar surface area, our methodology for the estimation of the alveolar surface area during the neonatal period might be useful to describe and monitor the pulmonary morbidity in those born extremely prematurely and could hold the potential to guide prognosis and clinical management.^38^ If our results were replicated in an independent cohort, the development of our index may provide useful grading information in this population of extremely preterm infants, and thus subsequently assist in identifying efficacious therapies, and better predict and prioritise those who require focused follow-up.^39^

In our study, we report very small alveolar tidal volumes (median 1.9 mL/kg), which might be inadequate for gas exchange according to traditional respiratory physiology. Recent studies have reported that effective carbon dioxide elimination might be possible with tidal volumes smaller that the dead space, possibly via spikes of fresh gas which penetrate through the dead space and create an interface of gas exchange in the conducting airways.^40^ The use of capnography to calculate dead space in premature ventilated infants has been recognised to have some technical limitations arising from high respiratory rates and small tidal volumes, which might cut short the expiration before the formation of an alveolar plateau.^27^ In our study, however, the CO_2_ sensor had a low apparatus dead space and its mainstream position in the respiratory circuit allowed for a faster response time even at high respiratory rates.

The adequacy of respiratory support could theoretically affect the aeration of the lungs and the subsequent calculations of the S_A_ in our study. From a clinical perspective, ventilation was deemed adequate as it achieved the target range of transcutaneous oxygen saturation and arterial carbon dioxide, and volume-targeted ventilation might have helped to avoid extremes of under- or overventilation. If, however, lung inflation and the level of respiratory support are not optimised, this might affect the reliability of the calculations. In this sense, the effect of the level of respiratory support on the S_A_ and the repeatability of the measurements over time should ideally be assessed in a separate future study.

Our study has strengths and some limitations. We reported a median pulmonary perfusion of 350 mL/min (compared to 314 mL/min in the baboon paper) and a median AaG of 599 mmHg (compared to 591 mmHg in the baboon paper).^13^ This high level of agreement between the human and the baboon studies highlights that the utilised regression equation is indeed transferable to human data. The baboon equation was derived from a group of baboons with a median weight of 0.37 kg compared to 0.805 kg in our included infants, and this size discrepancy might have an impact on the transferability of the baboon equation to the human infants. As our index, however, is meant to be an estimate rather than a true measurement of the S_A_, the size difference would not have affected the ability of the index to discriminate the pathological state of a need for home oxygen from not needing home oxygen. We have also reported that the adjusted S_A_ predicted the need for supplemental home oxygen with moderate accuracy and an area under the curve of 0.815. We have to consider, however, that this prediction happens very early in postnatal life (7 days) and discharge from neonatal care takes place at a much later age (the median age at discharge was 131 days in our cohort). The majority of this period is spent in intensive care with numerous possible further complications such as episodes of severe infection or life-threatening non-respiratory complications such as necrotising enterocolitis. Considering the above, we believe that the predictive ability of the adjusted S_A_ was remarkably high. A future study could measure the adjusted S_A_ before discharge or at 36 weeks postmenstrual age to predict later morbidity; this time point, however, would be more an assessment of severity rather than a prediction of neonatal outcomes.

Methodologically, we should also note that a certain degree of distorted lung architecture in BPD would cause diffusion limitation within the airway lumen rather than at the alveolar membrane level. This would be captured by a decrease in V_A_/Q, but does not strictly describe a decrease of the alveolar surface area. The relative contribution of this phenomenon, however, might not be particularly significant in this population, as the slope of the second phase of volumetric capnography (an index that describes abnormal gas mixing at the airways), has been reported to be similar in healthy term and ventilated preterm infants.^41^

We performed a comprehensive physiological assessment in a vulnerable cohort of infants. Previously described methods of calculating the V_A_/Q, respiratory dead space and focused echocardiography^13,20,26^ were combined with a recently validated animal model.^13,20,26^ We should acknowledge that the current cohort included only infants born extremely prematurely and all infants developed BPD; hence, the adjusted S_A_ index was unable to differentiate between those preterm infants who did and did not develop BPD. In our study, there was no association of the alveolar surface area (adjusted or unadjusted) with demographics at birth, such as gestational age and birth weight. This was an unexpected finding as lung growth would be expected to follow somatic growth and overall development. This lack of association might be explained by the narrow range of our included subjects (all less than 28 weeks of gestation). We selected this range, however, in order to focus on the ability of the biomarker to quantify clinical outcomes and to minimise the dilutional effect from the inclusion of larger and more mature infants in whom arrested alveolar growth would be less profound.

The echocardiographic findings in our study included a left-to-right direction of ductal shunt in all but one infants and a bidirectional shunt in the remaining one. Our calculations of V_A_/Q were, thus, not significantly affected by hypoxaemia resulting from cardiac right-to-left shunting. It is plausible that in the presence of significant pulmonary hypertension, some degree of atrial right-to-left shunting could have affected our calculations, but the diagnosis of pulmonary hypertension in premature infants would not be made before 28 days of life^42^ and our subjects were studied at 7 days. Finally, a significant proportion of our infants received medical or surgical treatment for PDA while recent literature has highlighted that expectant management is not inferior to PDA treatment with regard to the development of BPD.^43^ Furthermore, a multi-centre randomised trial of early treatment versus expectant management in extreme preterms,^44^ which was published very recently, also highlighted that expectant management was non-inferior to early treatment with respect to BPD.^45^

In conclusion, we have described a new, early biomarker of chronic respiratory disease in extreme prematurity, which was based on the pathophysiological mechanisms that explain gas exchange impairment in these infants. The adjusted alveolar surface area index predicted the need for the discharge of supplemental oxygen.

## Data Availability

The datasets generated and analysed during the current study are available from the corresponding author on reasonable request.
